# Detection of postoperative delirium by family and caregivers: Evaluation of the family confusion assessment method (FAM-CAM)

**DOI:** 10.1016/j.jclinane.2025.111963

**Published:** 2025-08-13

**Authors:** Angela M. Mickle, Bethany R. Tellor Pennington, Arbi Ben Abdallah, Wei Wang, Nan Lin, Jordan Oberhaus, Thaddeus P. Budelier, Daniel Park, Ben J. Palanca, Troy S. Wildes, Eva M. Schmitt, Sharon K. Inouye, Michael S. Avidan

**Affiliations:** a Department of Anesthesiology, Washington University School of Medicine, St. Louis, MO, United States of America; b Department of Statistics and Data Science, Washington University in St. Louis, MO, United States of America; c Department of Anesthesiology, University of Nebraska Medical Center, Omaha, NE, United States of America; d Department of Medicine, Beth Israel-Deaconess Medical Center, Hebrew SeniorLife, Harvard Medical School, Boston, MA, United States of America

**Keywords:** Delirium, Geriatric surgery, Confusion assessment method (CAM), Family confusion assessment method (FAM-CAM)

## Abstract

**Objective:**

The primary objective was to evaluate agreement between researchers’ and family members’ postoperative delirium assessment. The secondary objective was to assess the incidence of positive FAM-CAM after hospital discharge up to 30-days postoperatively.

**Methods:**

This was a pre-specified sub-study of two multicenter randomized controlled trials that evaluated interventions to prevent postoperative delirium in older adults undergoing major elective surgery. In the hospital, delirium was ascertained using the Confusion Assessment Method (CAM) long-form or the Confusion Assessment Method for the Intensive Care Unit (CAM-ICU), and structured chart review. Family members completed a Family Confusion Assessment Method (FAM-CAM) concurrent with researchers’ assessments in the afternoons on postoperative days 1–3. At the time of hospital discharge, a booklet of FAM-CAM surveys was provided to complete daily until postoperative day 30. Agreement between researcher-rated CAM/CAM-ICU and family-rated FAM-CAM was analyzed using Generalized Linear Mixed Model with repeated measures, and Bland-Altman analysis. Inter-rater reliability for each instrument was modeled using intraclass correlation coefficient (ICC). Overall agreement beyond chance between researcher’s assessment and the FAM-CAM was evaluated using repeated measure Cohen’s Kappa and sensitivity, specificity, and positive and negative predicted values. Post-discharge FAM-CAM data were summarized descriptively.

**Results:**

A total of 817 patients had 1349 concurrent delirium assessments. Postoperative delirium incidence by researchers’ assessment was 18.8 % and detection of delirium symptoms by FAM-CAM was 22.4 %. Analysis comparing delirium assessments showed there is an observed agreement beyond chance of 79.7 % with a kappa of 0.33 between the assessments by Generalized Linear Mixed Modeling with repeated measures, treating patients and raters as random effects, with FAM-CAM being more likely to report a positive delirium outcome. Assessment by features showed similar results. Both methods had an excellent degree of internal validity (CAM/CAM-ICU intraclass correlation =0.938, FAM-CAM intraclass correlation = 0.985). Repeated measures Cohen’s kappa indicated good overall agreement (kappa = 0.72 [95 % confidence interval, 0.63 to 0.81]). Of the 330 booklets, 133 (40.3 %) were returned. A total of 18 patients exhibited symptoms indicative of delirium based on the FAM-CAM assessment between hospital discharge and 30 days postoperatively. Out of these, 9 (50 %) had also been diagnosed with postoperative delirium during their hospitalization.

**Conclusion:**

This study demonstrated that family member completed FAM-CAM had acceptable agreement with researchers’ delirium assessments. Postoperative delirium symptoms were detected more frequently by family-administered FAM-CAM compared to delirium incidence identified by researcher assessments. Family members identified that some patients experienced delirium symptoms after hospital discharge.

## 1. Introduction

Postoperative delirium is a common complication following surgery, characterized by (i) an acute fluctuating change in mental status, (ii) inattention, and (iii) disorganized thinking or (iv) altered level of consciousness [1]. Postoperative delirium is associated with longer hospital stays, increased morbidity and mortality, and an increased financial burden on the healthcare system [2,3]. Multi-component non-pharmacological interventions such as light and sleep therapy, hydration and adequate nutrition, exercise, and cognitive therapy can mitigate delirium incidence, duration, severity, and long-term negative outcomes [4–6]. Consistent implementation of these interventions, combined with early detection of delirium, are essential to improve patient outcomes in older adults following major surgery.

While there are no “gold standard” tools for detecting postoperative delirium, the Confusion Assessment Method (CAM) [7] and the Confusion Assessment Method for the Intensive Care Unit (CAM-ICU) [8] are well validated delirium assessment tools but require training and are not usually translated into standard care strategies for delirium detection. Although there is a plethora of literature on delirium in the postoperative period, it often goes undiagnosed or unrecognized [9]. Often, healthcare professionals are not well trained to look for symptoms of delirium, lack effective screening tools, or delirium diagnosis is missed due to its fluctuating nature [9]. Family members, caregivers, or friends may recognize unusual symptoms not characteristic of their family members that the healthcare professionals may miss [10]. Therefore, engaging caregivers, family, or friends is essential to improve early identification of delirium symptoms in the postoperative period when patients are the most vulnerable [11].

The Family Confusion Assessment Method (FAM-CAM) [12] is an 11-question delirium screening tool developed for this purpose. Based on the scoring of the CAM, it is designed to increase caregivers’ awareness of delirium symptoms, enabling families to bring these symptoms to medical attention and facilitating the diagnostic and therapeutic process [12]. In preliminary research, the FAM-CAM has been shown to have good agreement with the CAM [12–14] and has been applied across various settings, including the community, emergency department, ICU, skilled nursing centers, intermediate and post-acute care settings in multiple countries [13–19] While available evidence supports the FAM-CAM’s strong performance in detecting delirium in adults across these settings, studies specifically examining its performance in postoperative hospital settings and after hospital discharge are limited [17,20].

Postoperative delirium during hospitalization after major surgery has been well characterized in different patient populations, but there is little research on delirium following hospital discharge [21]. This is especially relevant because there may be less active monitoring, care, and vigilance at patients’ homes. With the large number of older adults undergoing major elective surgery and many of them discharged home early, it could be helpful if family caregivers are able to assess for delirium symptoms, especially after hospital discharge [22,23]. Identification of such symptoms could prompt timely follow-up and a formal assessment by a clinician, potentially leading to clinically relevant interventions.

The first objective of this study was to assess agreement between the researchers’ concurrent postoperative delirium assessment (CAM/CAM-ICU) and the FAM-CAM in older adults undergoing major elective surgery during postoperative days 1–3. The second objective was to characterize whether this patient population experiences delirium symptoms resulting in a positive FAM-CAM after hospital discharge up to 30 days postoperatively, by family report.

## 2. Methods

### 2.1. Recruitment

Patients were recruited to the Electroencephalography Guidance of Anesthesia to Alleviate Geriatric Syndromes (ENGAGES) study or the Prevention of Delirium and Complications Associated with Surgical Treatments (PODCAST) study [24–27]. This was a pre-specified sub-study of both the ENGAGES and POCAST studies [24,25]. Patients eligible for the ENGAGES study were 60 years or older, able to give informed consent, and scheduled for major elective surgery under general anesthesia with a potent volatile anesthetic, requiring at least a 2-day postoperative hospital stay [25]. Patients eligible for the PODCAST study were 60 years or older, able to give informed consent, and undergoing major open cardiac surgery or major non-cardiac surgeries under general anesthesia [24]. All patients provided written informed consent. This sub-study was approved by the Washington University Institutional Review Board. This manuscript follows the STROBE reporting guidelines for observational studies [28].

### 2.2. Delirium assessment training

Research staff completed a structured training regimen prior to completing any postoperative delirium assessments as previously described [25,29]. Research members also were instructed on training family members to complete the FAM-CAM using standardized training materials and procedures, reviewing *The Family Confusion Assessment Method (FAM-CAM) Instrument and Training Manual,* and completing an in-person training session by a researcher trained in delirium assessments [12]. Family members were instructed on completing the FAM-CAM per the instruction guide provided in the training manual. Family members or close friends were asked to be objective when completing the FAM-CAM; any key features of delirium that were present as well as changes from usual preoperative behavior were reported and marked as present. Only individuals living with the patient, or those who had seen the patient at least once a month and knew the patient well enough to report mental and physical abilities about the patient were asked to complete the FAM-CAM survey.

### 2.3. Delirium assessments in the hospital

After surgery, patients were assessed for postoperative delirium by trained research staff per study protocols [24,25]. For the ENGAGES study, patients were assessed daily on postoperative days (POD) 1–5 in the afternoon using the CAM or CAM-ICU, and with structured delirium chart review as the combined approach increases the sensitivity of delirium detection and provides a more comprehensive detection of delirium [30–32]. Patients in the PODCAST study were assessed on POD 1–3 in both the morning and afternoon using the CAM or CAM-ICU. Delirium was assessed using the CAM preferentially in both studies. The CAM-ICU was used for patients who were unable to speak (e.g. tracheal tube or tracheostomy in place) and who were not sedated beyond a Richmond Agitation and Sedation Scale > − 4, or for those patients who refused the full CAM assessment. The CAM assessment has been validated in older adults across multiple care settings, including the intensive care unit, emergency department, and outpatient clinics [33,34]. Additionally, the CAM-ICU has been well studied in critically ill patients [8,35,36]. Both the CAM and the CAM–ICU have exhibited sensitivity of 94–100 %, specificity of 89–95 % compared to a geriatric psychiatrist using Diagnostic and Statistical Manual of Mental Disorders-IV criteria [37]. For the CAM and CAM-ICU, standard algorithm-based scoring criteria was applied as originally validated [7,8]. For the structured delirium chart review in the ENGAGES study, trained staff blinded to the intervention of the respective study and to the CAM/CAM-ICU assessment results reviewed the patients’ medical records including nurses’ notes, progress notes, consult notes, specialist reports, ICD codes, cognitive assessments and nursing delirium assessments for any evidence of acute confusional state. Any evidence including but not limited to words such as delirium, mental status change, inattention, disorientation, hallucinations, agitation, or inappropriate behavior was documented from the chart verbatim for each day POD 1–5 and sent to an external expert panel for adjudication [25]. For the structured chart review, published methodology and criteria for retrospective identification of delirium, as described in prior validation studies, was used [30,31].

In both studies, if a relative was present during the researcher’s assessment, they were asked to complete the FAM-CAM prior, during, or shortly after the researcher’s delirium assessment. The FAM-CAM has been validated in older adults who are cognitively intact as well as in individuals with dementia both in clinic and hospital settings. The FAM-CAM has shown to have good agreement with the CAM (sensitivity 88 % (95 % CI 47–99 %), specificity 98 % (95 % CI 86–100 %) as well as with rigorous assessments using Diagnostic and Statistical Manual of Mental Disorders-IV criteria [12] (sensitivity 75 % (95 % CI 35–95 %), specificity 91 % (95 % CI 74–97 %). The FAM-CAM was independently reviewed and scored by a separate blinded research staff member according to previously validated evaluation criteria [12]. For the primary objective, patients with concurrent CAM/CAM-ICU and FAM-CAM assessments completed in the afternoon of POD 1–3 from either study were included in the analysis.

### 2.4. Delirium assessments after hospital discharge

Prior to discharge or on the last day of assessments, family members of 330 participants in the ENGAGES study were given a FAM-CAM booklet and instructed to complete daily FAM-CAM surveys up to POD 30 and return the booklet by mail. A total of 1213 participants were able to complete at least one delirium assessment POD 1–5 (i.e. not sedated or deceased). Of those assessable, 883 were not provided FAM-CAM booklets for one of the following reasons: the patient was discharged early and missed providing a booklet, the patient did not have family members present at the time of the last delirium assessment, the patient was to be discharged to a nursing facility where no family member was available, the patient lived alone and did not have family members to complete a booklet, or the family declined to complete. There were no researcher’s delirium assessments or chart review performed after POD 5, during the 30-day follow-up period. For the second objective, both the researcher’s CAM/CAM-ICU and structured chart review on POD 1–5 from the ENGAGES study were used to assess the trajectory of postoperative in-hospital delirium.

### 2.5. Statistical analysis

#### 2.5.1. Agreement between researchers’ and family members’ delirium assessments (Primary objective)

Characteristics are presented as descriptive statistics for those patients who had concurrent CAM/CAM-ICU and FAM-CAM assessments. To be considered positive for delirium, acute change, inattention, and either disorganized thinking or altered level of consciousness are required. The features of delirium including acute change, inattention, either disorganized thinking or altered level of consciousness, and overall delirium outcomes were compared between CAM/CAM-ICU and FAM-CAM assessments. Disorganized thinking or altered level of consciousness were combined based on the scoring algorithm for the FAM-CAM [12]. Agreement between the CAM/CAM-ICU and FAM-CAM was analyzed using the Generalized Linear Mixed Model (GLMM) with repeated measures, treating patients and raters as random effects. Bland-Altman analysis was also conducted to assess agreement between CAM/CAM-ICU and FAM-CAM. Inter-rater reliability for each instrument was modeled using intraclass correlation coefficient (ICC). Agreement of each delirium feature (acute onset or fluctuation, inattention, disorganized thinking/altered level of consciousness) was compared between researcher’s CAM/CAM-ICU and FAM-CAM assessment using the GLMM [38]. Overall agreement beyond chance between the two instruments was evaluated using repeated measure Cohen’s Kappa and sensitivity, specificity, and positive and negative predicted values. Using the research-rated CAM/CAM-ICU as the reference standard, the adjusted sensitivity, specificity, positive predictive value (PPV) and negative predictive value (NPV) was calculated [39–41]. A total of 7 FAM-CAM surveys were missing an answer to the question about inattention. There was no imputation of missing data for any item in the FAM-CAM. Data analysis was completed using SAS 9.4 (SAS Institute, Cary, North Carolina) and R statistical software (3.4.2) using the ‘psych’ package.

#### 2.5.2. FAM-CAM after hospital discharge (Secondary objective)

Patient characteristics are presented as descriptive statistics for participants with concurrent CAM/CAM-ICU and FAM-CAM surveys and patients whose family members were given a FAM-CAM booklet. Data are segregated according to whether a booklet was returned. Descriptive characteristics for patients who were positive for delirium according to FAM-CAM booklets are presented.

## 3. Results

### 3.1. Primary objective

**Fig. 1.**
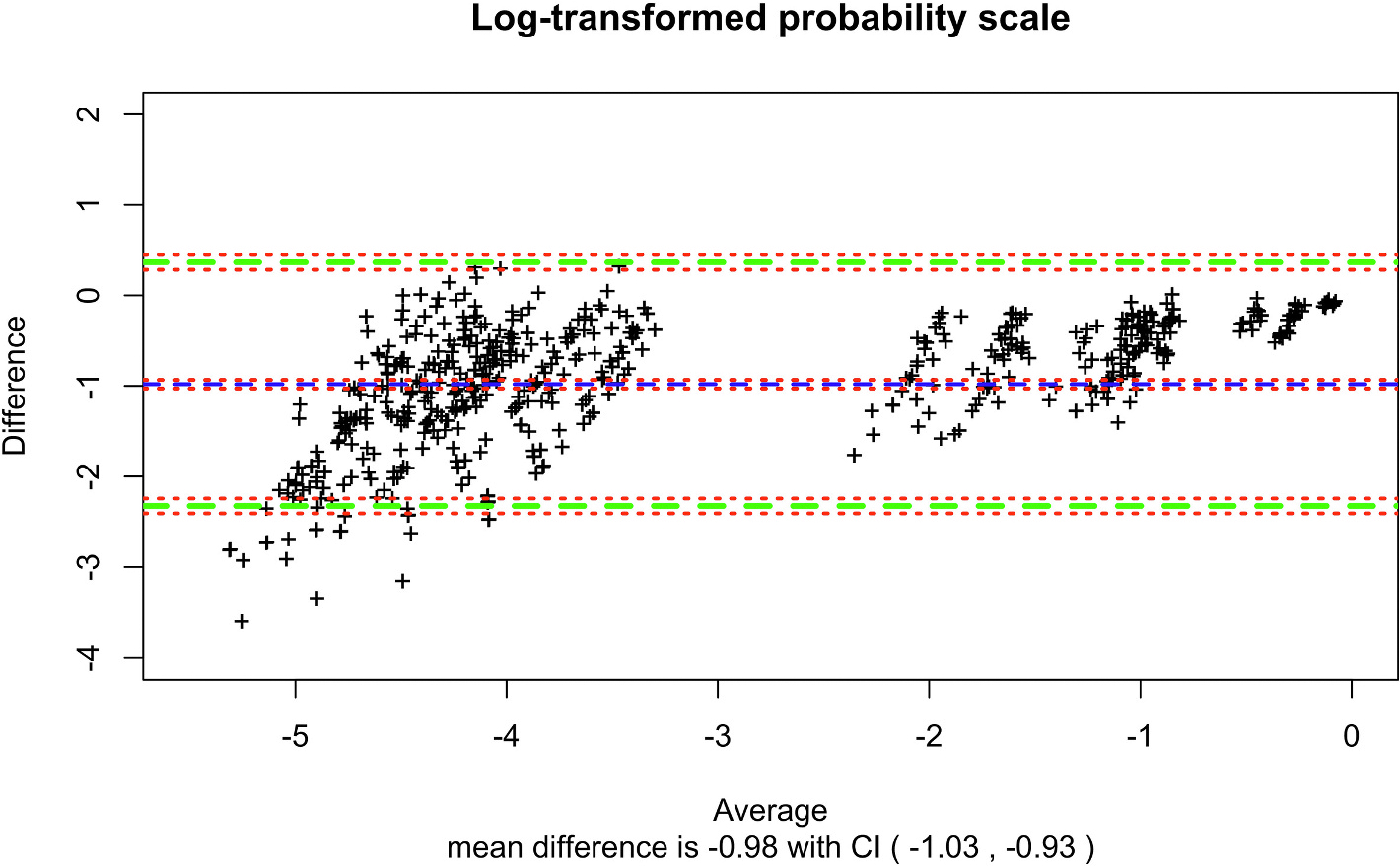
Bland Altman Plot for the log-transformed probability scale. Each ‘+’ represents a patient. The x-axis is the average of log (probabilities of a positive outcome (delirium)) measured by CAM and FAM-CAM. The y-axis is the difference between log (probabilities of a positive outcome (delirium)) measured by CAM and FAM-CAM. For example, a point in the plot with y-value −0.1 means, for that patient, log (probabilities of a positive outcome (delirium)) based on method CAM is 0.1 lower than that based on method FAM-CAM. In other words, the ratio of probabilities of positive outcome (delirium) between CAM and FAM-CAM is e^−0.1^ = 0.9. The paired green dashed lines plot the 95 % agreement limits, which also covers the zero difference. As expected, 95.4 % of the points lie within ±2 standard deviations of the mean difference. The blue dashed line shows the mean difference. The mean difference is −0.98 [95 % CI, −1.03 to −0.93], which indicates that the probability of a positive outcome (delirium) measured by FAM-CAM is on average e^0.98^ = 2.7 times the probability by CAM. (For interpretation of the references to colour in this figure legend, the reader is referred to the web version of this article.)

A total of 817 patients (Table 1) had at least one concurrent CAM/CAM-ICU and FAM-CAM survey completed between POD 1 through 3. In these patients, there were 1349 paired CAM/CAM-ICU and FAM-CAM assessments. Over half of the FAM-CAM assessments completed in the hospital were completed by patients’ spouses or partners (Table 2). The overall postoperative delirium incidence by researchers’ ascertainment defined by at least one positive delirium assessment (CAM or CAM-ICU) in these patients was 18.8 % (154/817), while the overall detection of possible delirium symptoms by the FAM-CAM survey defined by at least one positive FAM-CAM was 22.4 % (183/817). The sensitivity and specificity comparing the FAM-CAM with the researchers’ assessments (CAM/CAM-ICU) was 53.3 % (95 % CI =0.45 to 0.61) and 87.8 % (95 % CI =0.86 to 0.90) with a PPV of 35.5 % (95 % CI =0.29 to 0.42) and a NPV of 93.8 % (95 % CI =0.93 to 0.95). According to the GLMM model, there was a significant difference in overall agreement in delirium outcome between concurrent paired CAM/CAM-ICU and FAM-CAM assessments (*p* = 0.0100) (Supplement 1). An observed agreement beyond chance of 79.7 % with a kappa of 0.33 between the assessments was found, with FAM-CAM being more likely to report a positive delirium outcome. There were also significant differences in agreement between the following delirium features: acute change (*p* = 0.0158), inattention (*p* ≤0.0001), and disorganized thinking/altered level of consciousness (p ≤0.0001). The sensitivity and specificity for acute change was 55.5 % (95 % CI =51.9 to 59.0) and 71.5 % (95 % CI = 67.9 to 75.1) with a PPV of 70.4 % (95 % CI =66.7 to 74.1) and a NPV of 56.7 % (95 % CI =53.2 to 60.2). The sensitivity and specificity for inattention was 26.4 % (95 % CI =22.7 to 30.0) and 88.4 % (95 % CI = 86.2 to 90.7) with a PPV of 62.3 % (95 % CI =56.2 to 68.5) and a NPV of 62.3 % (95 % CI =59.4 to 65.1). The sensitivity and specificity for disorganized thinking/altered level of consciousness was 83.6 % (95 % CI =78.1 to 89.2) and 53.3 % (95 % CI = 50.5 to 56.2) with a PPV of 20.6 % (95 % CI =17.9 to 23.7) and a NPV of 95.7 % (95 % CI =94.2 to 97.3). Testing the modeled intraclass correlation, both methods found raters had an excellent degree of agreement (CAM ICC = 0.938, FAM-CAM ICC = 0.985). Bland Altman analysis indicated the probability of a positive FAM-CAM was greater than the researchers’ assessment (Fig. 1). Overall delirium determination by CAM/CAM-ICU and FAM-CAM indicated good agreement beyond chance by repeated measures Cohen’s Kappa (0.72 [95 % CI = 0.63 to 0.81]). Cohen’s kappa for agreement beyond chance for individual features were 0.47 (95 % CI = 0.42 to 0.52) for acute change, 0.07 (95 % CI = 0.04 to 0.10) for inattention and 0.14 (95 % CI = 0.10 to 0.17) for disorganized thinking/altered level of consciousness. Adjusted sensitivity and specificity took into consideration the bias induced by an imperfect gold standard using previously published sensitivities and specificities of each measure [42,43]. The pooled sensitivities and specificity for CAM/CAM-ICU were 82 % [95 % CI = 0.69 to 0.91] and 99 % [95 % CI = 0.87 to 1.00]. Under the conditionally independent assumption, the adjusted specificity of FAM-CAM was 60 %, and the adjusted specificity was 85 %. The PPV was 57 % [95 % CI =0.48 to 0.66), and the NPV was 84 % [95 % CI = 0.81 to 0.86].

### 3.2. Secondary objective

**Fig. 2.**
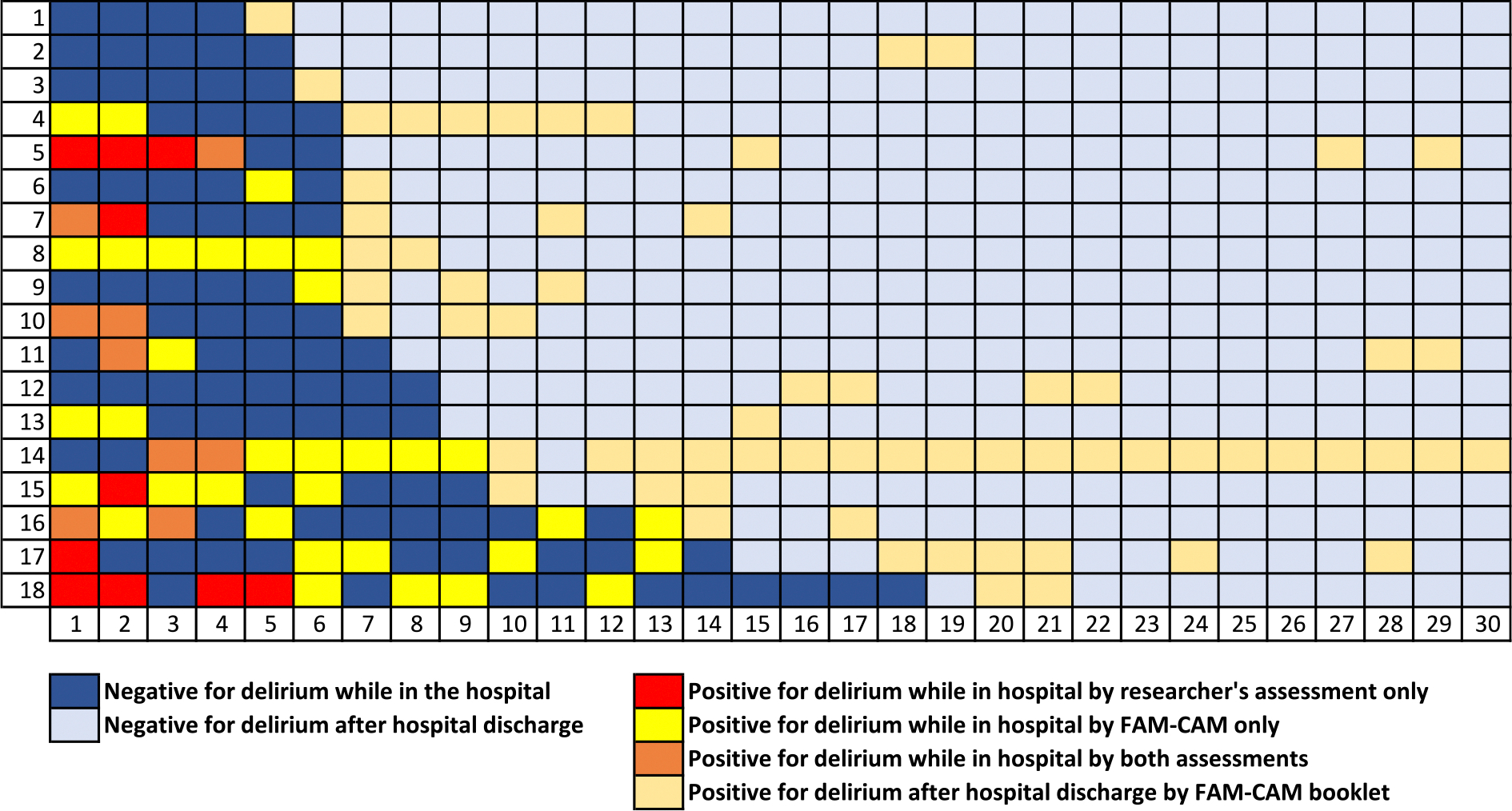
Delirious Patients by FAM-CAM Booklet. Trajectory of the 18 patients who were found to be positive by FAM-CAM booklet during POD 1–30. Figure shows when the patients were found to be positive by researchers’ delirium assessment, FAM-CAM and when the assessments agreed. Note: patients were assessed by researchers until POD 5 while in the hospital.

Of the family members of 330 patients, 130 (39.4 %) returned a booklet with at least one day complete. Baseline characteristics of patients whose families returned a booklet compared to non-responders are presented in Table 3. The majority of patients whose family members returned a booklet were male, Caucasian and had at least a high school degree or equivalent, or a higher level of education. A total of 18 patients had a positive FAM-CAM at least once between hospital discharge and 30 days postoperatively. Out of these, 9 (50 %) had postoperative delirium according to CAM, CAM-ICU, or delirium chart review assessments during their hospital stay (Fig. 2). Baseline characteristics of the patients who were positive for delirium after discharge compared to patients who were negative are presented in Table 4.

## 4. Discussion

The main finding of this study is that family members can use the FAM-CAM assessment to identify symptoms of delirium, with results showing acceptable agreement with the CAM or CAM-ICU assessments, a finding also supported previously [20]. These results suggest that the FAM-CAM, designed to enhance early recognition of delirium symptoms, is a practical tool for non-clinicians to use in the postoperative period. It can aid in the detection of delirium symptoms after major surgery in order to effectively supplement the CAM/CAM-ICU and ensure timely medical intervention. This study also raises the possibility that surgical patients may be experiencing delirium after hospital discharge, approximately half of whom were not diagnosed with postoperative delirium during their hospital stay.

While the FAM-CAM had a higher likelihood of detecting possible delirium symptoms compared to the researchers’ assessment, the sensitivity and PPV were lower than what has been previously reported [14]. This may be due to differences in how and when assessments were conducted, the fluctuating nature of delirium, variability in how family caregivers interpret symptoms, or how well they were trained. Notably, the FAM-CAM captures behavior over a 24-h period, whereas clinical tools like the CAM or CAM-ICU rely on brief, point-in-time evaluations. This extended window may allow family members to notice subtle or transient symptoms that clinicians could miss. Despite the lower sensitivity and PPV, the specificity, NPV, and overall agreement between the FAM-CAM and the researchers’ assessments were good. This suggests that while family caregivers may over-identify symptoms, their reports are generally reliable for ruling out delirium. These findings highlight that, without a definitive gold standard for delirium diagnosis, a single assessment method may be inadequate. A combined approach using both clinical assessments and family-reported tools like the FAM-CAM may provide a more complete picture. Family caregivers who are familiar with the patient’s baseline cognitive and physical functioning, particularly those who have seen them at least once in the last month and received delirium education, may improve early detection and timely intervention after major surgery. Their involvement could also aid in identifying subsyndromal delirium [44,45], which may otherwise go undetected. Effective communication and collaboration between clinicians and family caregivers are likely to play a key role in improving the identification and management of postoperative delirium.

Our findings suggest that delirium symptoms may also be present after hospital discharge in some surgical patients who were not identified as delirious during hospitalization. However, these results should be interpreted cautiously given the limitations in follow-up data and lack of a clinical reference standard. The different study designs may have influenced the incidence of delirium found during hospitalization. The PODCAST study assessed delirium twice a day for three days, while the ENGAGES study assessed delirium only in the afternoon for five days. Additionally, both studies were interventional trials and may have influenced delirium outcomes or caregivers’ perception of delirium, which may have influenced their reporting. Future research is needed to validate the FAM-CAM in post-discharge or home-based settings using standardized clinical assessments, to better understand the incidence and risk factors for post-discharge delirium, and to develop effective prevention and management strategies.

This study has limitations. First, there is no objective biomarker or gold standard for delirium. We did not compare the findings of the CAM and CAM-ICU separately against the FAM-CAM, as this was not feasible. Each patient was assessed with either the CAM or CAM-ICU daily, but not both. However, we and others have previously shown that the long CAM form is a reliable instrument in verbal awake patients with excellent agreement between raters while the CAM-ICU is reliable in ventilated or non-verbal patients [29,36]. Thus, these tools are reasonable instruments to use as a reference for other candidate assessments, as we did in this study. Second, among those participants who were accessible, many were not provided FAM-CAM booklets for the various reasons listed above. Consequently, the lack of data from this subgroup could lead to underestimation or inaccurate conclusions regarding the prevalence and relevance of delirium symptoms in the overall population. Furthermore, the inability to comprehensively report missing data for patients without concurrent researcher delirium assessments and FAM-CAM surveys, or for those not administered a FAM-CAM, may introduce selection and information bias, potentially affecting the accuracy and generalizability of the findings. Third, to maintain blinding and prevent bias in the scoring of the CAM/CAM-ICU, assessors who completed the delirium interview did not review the FAM-CAM after it was completed. Therefore, some data were missing, and family members may have misinterpreted questions when completing the FAM-CAM survey. Additionally, family caregiver demographic information was not collected, which could lead to potential bias in reporting. The family members were asked about the patient’s behavior the previous 24 h, but it is unknown how long or often the family members were with the patient before completing the assessment. No answers on the assessments were changed. However, some family members, instead of marking ‘yes’ for features like excessive drowsiness, noted in comments that they viewed such symptoms as normal for someone recovering from surgery and taking medications. This could account for some of the discrepancies between the concurrent CAM/CAM-ICU and FAM-CAM, not only in the presence of the features, but also the overall assessment. Fourth, of the 330 FAM-CAM booklets sent home, only 150 were returned, which may be influenced by both selection and responder bias. The low response rate and the absence of a gold standard clinical assessment after discharge limit any definitive conclusion that can be made regarding the proportion of individuals who may have experienced delirium post discharge. Caution should be applied when interpreting these secondary findings and future studies should be conducted using validated clinical assessments as a reference standard to assess this matter. Fifth, the FAM-CAM booklet is an adaptation of the assessment tool, where family members were only instructed on the instrument once rather than at each time point. Sixth, the home is a very different environment from the hospital setting, which may impact family member responses. Future studies should further explore reliability of the FAM-CAM completed outside of the hospital setting and incorporate item-level data collection over time to better capture and analyze how relatives’ observations of delirium symptoms change. Seventh, no delirium chart review was conducted for the PODCAST study. Although delirium was assessed twice a day, delirium is fluctuating in nature and therefore it is possible that episodes of delirium may have been missed. Lastly, dementia incidence was not formally recorded in the parent studies. While some participants may have had a history of dementia, this was likely uncommon, as all underwent cognitive screening and provided informed consent. The absence of confirmed dementia diagnosis may have influenced how delirium symptoms were perceived and reported.

In conclusion, this study demonstrates that involving family members can be practically implemented, with the understanding that clear instructions for completing the FAM-CAM are necessary to ensure optimal reporting. Family engagement could be bolstered by reaching out to family members prior to surgery and educating them on risk factors for and symptoms of delirium [46,47]. By early identification of delirium symptoms, family members could potentially partner with the healthcare team to enhance the ability of the team to diagnose delirium and to implement preventative strategies and safety measures to prevent known complications of postoperative delirium [48]. Future studies should clarify the incidence of postoperative delirium following discharge, validate the FAM-CAM in this setting, identify patients at highest risk, and develop strategies to prevent its negative consequences.

## Supplementary Material

MMC1

Appendix A. Supplementary data

Supplementary data to this article can be found online at https://doi.org/10.1016/j.jclinane.2025.111963.

## Figures and Tables

**Table 1:** Baseline Characteristics: Patients with Concurrent Assessments.

Baseline Characteristics	Total (*n* = 817 patients)	Delirious (*n* = 154 patients)	Non-Delirious (*n* = 663 patients)

Age mean ± std	70.2 ± 6.8	71.4 ± 6.6	69.9 ± 6.8
Sex n (%)			
Male	461 (56.4)	86 (55.8)	375 (56.6)
Female	356 (43.6)	68 (44.2)	288 (43.4)
Race n (%)			
Caucasian	727 (89.0)	132 (85.7)	595 (89.7)
African American	52 (6.4)	16 (10.4)	36 (5.4)
Other	27 (3.3)	1 (0.6)	26 (3.9)
Not Reported	11 (1.4)	5 (3.3)	6 (0.9)
Ethnicity n (%)			
Non-Hispanic	762 (93.3)	145 (94.2)	617 (93.1)
Hispanic	8 (1.0)	0 (0.0)	8 (1.2)
Not reported	47 (5.7)	9 (5.8)	38 (5.7)
Level of education n (%)			
Less than HS degree	77 (9.4)	15 (9.7)	62 (9.4)
HS degree or equivalent	221 (27.1)	41 (26.6)	180 (27.2)
Some College/Degree	329 (40.3)	60 (39.0)	269 (40.6)
Professional	116 (14.2)	18 (11.7)	98 (14.8)
Not reported	74 (9.1)	20 (13.0)	54 (8.1)
Surgery Type n (%)			
Cardiac	323 (39.5)	69 (44.8)	254 (38.3)
Gastrointestinal	52 (6.4)	5 (3.25)	47 (7.1)
Gynecologic	15 (1.8)	0 (0.0)	15 (2.3)
Hepatobiliary-Pancreatic	114 (14.0)	27 (17.5)	87 (13.1)
Orthopedic/Spine	3 (0.4)	1 (0.7)	2 (0.3)
Thoracic	61 (7.5)	11 (7.1)	50 (7.5)
Urologic	67 (8.2)	5 (3.3)	62 (9.4)
Vascular	80 (9.8)	21 (13.6)	59 (8.9)
Other	102 (12.5)	15 (9.7)	87 (13.1)

Baseline characteristics of patients who had concurrent researchers’ delirium assessment and Family Confusion Assessment Method (FAM-CAM) surveys completed in the immediate postoperative period (POD 1–3). Patients in this table were noted delirious by the researchers’ delirium assessment, Confusion Assessment Method (CAM) or Confusion Assessment Method for the Intensive Care Unit (CAM-ICU).

Std = standard deviation.

**Table 2:** Family Confusion Assessment Method (FAM-CAM) Survey.

Relationship n (%)	Total Assessments (*n* = 1349 assessments)	Delirious (*n* = 150 assessments)	Non-Delirious (*n* = 1199 assessments)

Spouse/Partner	782 (58.0)	83 (55.3)	699 (58.3)
Son/Daughter	389 (28.8)	45 (30.0)	344 (28.7)
Brother/Sister	64 (4.7)	9 (6.0)	55 (4.6)
Mother/Father	5 (0.4)	0 (0.0)	5 (0.4)
Son/daughter-in-law	15 (1.1)	1 (0.7)	14 (1.2)
Brother/sister-in-law	13 (1.0)	3 (2.0)	10 (0.8)
Grandchild	10 (0.7)	1 (0.7)	9 (0.8)
Close friend	36 (2.7)	2 (1.3)	34 (2.8)
Other	25 (1.9)	5 (3.3)	20 (1.7)
Not Reported	10 (0.7)	1 (0.7)	9 (0.8)

Relationship of those who completed the Family Confusion Assessment Method (FAM-CAM) survey delineated delirium status according to researchers’ assessment, Confusion Assessment Method (CAM) or Confusion Assessment Method for the Intensive Care Unit (CAM-ICU). Totals are by the number concurrent researchers’ delirium assessment and FAM-CAM’s during POD 1–3.

**Table 3:** Baseline Characteristics: Family Confusion Assessment Method (FAM-CAM) Booklet Patients.

Baseline Characteristics	Total (*n* = 330 patients)	Returned booklets (*n* = 130 patients)	Did not return booklets (*n* = 200 patients)

Age mean ± std	70.5 ± 7.2	70.9 ± 7.5	70.3 ± 6.9
Sex n (%)			
Male	192 (58.2)	80 (61.5)	112 (56.0)
Female	138 (41.8)	50 (38.5)	88 (44.0)
Race n (%)			
Caucasian	314 (95.2)	127 (97.7)	187 (93.5)
African American	15 (4.5)	3 (2.3)	12 (6.0)
Other	1 (0.3)	0 (0.0)	1 (0.5)
Ethnicity n (%)			
Non-Hispanic	326 (98.8)	129 (99.2)	197 (98.5)
Hispanic	4 (1.2)	1 (0.8)	3 (1.5)
Level of education n (%)			
Less than HS degree	25 (7.6)	12 (9.2)	13 (6.5)
HS degree or equivalent	105 (31.8)	40 (30.8)	65 (32.5)
Some College/Degree	128 (38.8)	46 (35.4)	82 (41.0)
Professional	43 (13.0)	17 (13.1)	26 (13.0)
Unreported	29 (8.8)	15 (11.5)	14 (7.0)
Surgery Type n (%)			
Cardiac	137 (41.5)	53 (40.8)	84 (42.0)
Gastrointestinal	24 (10.4)	9 (7.0)	15 (7.5)
Gynecologic	10 (4.3)	9 (2.3)	1 (0.5)
Hepatobiliary-Pancreatic	56 (24.3)	26 (20.0)	30 (15.0)
Thoracic	27 (11.7)	14 (10.8)	13 (6.5)
Urologic	24 (10.4)	5 (3.9)	19 (9.5)
Vascular	31 (13.5)	13 (10.0)	18 (9.0)
Other	27 (11.7)	7 (5.4)	20 (10.0)

Baseline characteristics of patients whose family returned the Family Confusion Assessment Method (FAM-CAM) booklet with at least one FAM-CAM survey completed compared to patients whose family members did not return a booklet.

Std = standard deviation.

**Table 4:** Baseline Characteristics: Delirious Patients by Family Confusion Assessment Method (FAM-CAM) Booklet.

Baseline Characteristics	Total (n = 130 patients)	Delirious (*n* = 18 patients)	Non-Delirious (*n* = 112 patients)

Age mean ± std	70.9 ± 7.5	68.8 ± 6.9	71.2 ± 7.5
Sex n (%)			
Male	80 (61.5)	12 (66.7)	68 (60.7)
Female	50 (38.5)	6 (33.3)	44 (39.3)
Race n (%)			
Caucasian	127 (97.7)	17 (94.4)	110 (98.2)
African American	3 (2.3)	1 (5.6)	2 (1.8)
Ethnicity n (%)			
Non-Hispanic	129 (99.2)	18 (100.0)	111 (99.1)
Hispanic	1 (0.8)	0 (0.0)	1 (0.9)
Level of education n (%)			
Less than HS degree	12 (9.3)	1 (5.6)	11 (9.8)
HS degree or equivalent	40 (30.8)	6 (33.3)	34 (30.4)
Some College/Degree	46 (35.4)	4 (22.2)	42 (37.5)
Professional	17 (13.1)	5 (27.8)	12 (10.7)
Not reported	15 (11.5)	2 (11.1)	13 (11.6)
Surgery Type n (%)			
Cardiac	53 (40.8)	5 (27.8)	48 (42.9)
Gastrointestinal	9 (6.9)	2 (11.1)	7 (6.3)
Gynecologic	3 (2.3)	0 (0.0)	3 (2.7)
Hepatobiliary-Pancreatic	26 (20.0)	3 (16.7)	23 (20.5)
Thoracic	14 (10.8)	3 (16.7)	11 (9.8)
Urologic	5 (3.9)	2 (11.1)	3 (2.7)
Vascular	13 (10.0)	2 (11.1)	11 (9.8)
Other	7 (5.4)	1 (5.6)	6 (5.4)

Baseline characteristics of those patients who were positive by Family Confusion Assessment Method (FAM-CAM) booklet after hospital discharge compared to patients who were negative by FAM-CAM booklet.

Std = standard deviation.

## Data Availability

The datasets generated during and/or analyzed during the current study are available from the corresponding author on reasonable request. The ENGAGES and PODCAST trials are registered at clinicaltrials.gov: NCT02241655, NCT01690988.

## HIGHLIGHTS

- The FAM-CAM is a useful tool to detect delirium symptoms postoperatively in the hospital and after hospital discharge.
- Some surgical patients may show delirium symptoms after discharge, despite no delirium identified during hospitalization.
- Family members can help detect delirium symptoms after hospital discharge when other methods are less practical.

## Abbreviations:

CAM: Confusion Assessment Method
CAM-ICU: Confusion Assessment Method for the Intensive Care Unit
ENGAGES: Electroencephalography Guidance of Anesthesia to Alleviate Geriatric Syndromes
FAM-CAM: Family Confusion Assessment Method
GLMM: Generalized Linear Mixed Modeling
ICC: Intraclass correlation coefficient
POD: Postoperative Day
PODCAST: The Prevention of Delirium and Complications Associated with Surgical Treatments

## References

[1] European Delirium Association, American Delirium Society. The DSM-5 criteria, level of arousal and delirium diagnosis: inclusiveness is safer. BMC Med 2014;12:141. 25300023 10.1186/s12916-014-0141-2 PMC4177077

[2] Leslie DL, Inouye SK. The importance of delirium: economic and societal costs. J Am Geriatr Soc 2011;59(Suppl. 2):S241–3. 22091567 10.1111/j.1532-5415.2011.03671.x PMC3415302

[3] Marcantonio ER. Postoperative delirium: a 76-year-old woman with delirium following surgery. JAMA 2012;308:73–81. 22669559 10.1001/jama.2012.6857 PMC3604975

[4] Hshieh TT, Yue J, Oh E, Puelle M, Dowal S, Travison T, Effectiveness of multicomponent nonpharmacological delirium interventions: a meta-analysis. JAMA Intern Med 2015;175:512–20. 25643002 10.1001/jamainternmed.2014.7779 PMC4388802

[5] Lundström M, Edlund A, Karlsson S, Brännström B, Bucht G, Gustafson Y. A multifactorial intervention program reduces the duration of delirium, length of hospitalization, and mortality in delirious patients. J Am Geriatr Soc 2005;53:622–8. 15817008 10.1111/j.1532-5415.2005.53210.x

[6] O’Mahony R, Murthy L, Akunne A, Young J, Guideline Development Group. Synopsis of the National Institute for health and clinical excellence guideline for prevention of delirium. Ann Intern Med 2011;154:746–51. 21646557 10.7326/0003-4819-154-11-201106070-00006

[7] Inouye SK, van Dyck CH, Alessi CA, Balkin S, Siegal AP, Horwitz RI. Clarifying confusion: the confusion assessment method. Ann Intern Med 1990;113:941–8. 2240918 10.7326/0003-4819-113-12-941

[8] Ely EW, Margolin R, Francis J, May L, Truman B, Dittus R, Evaluation of delirium in critically ill patients: validation of the confusion assessment method for the intensive care unit (CAM-ICU). Crit Care Med 2001;29:1370–9. 11445689 10.1097/00003246-200107000-00012

[9] Titlestad I, Haugarvoll K, Solvang S-EH, Norekvål TM, Skogseth RE, Andreassen OA, Delirium is frequently underdiagnosed among older hospitalised patients despite available information in hospital medical records. Age Ageing 2024:53. 10.1093/ageing/afae006. PMC10859244 38342753

[10] Golden BP, Kaiksow FA, Kind AJ. Partnering is paramount: engaging care partners to improve delirium identification. WMJ 2024;123:163–5. 39024135 PMC11755429

[11] Rosgen B, Krewulak K, Demiantschuk D, Ely EW, Davidson JE, Stelfox HT, Validation of caregiver-centered delirium detection tools: a systematic review. J Am Geriatr Soc 2018;66:1218–25. 29671281 10.1111/jgs.15362

[12] Inouye SK, Puelle MR, Saczynski JS, Steis MR. The family confusion assessment method (FAM-CAM): Instrument and training manual. Boston, MA: Hospital Elder Life Program; 2011.

[13] Flanagan NM, Spencer G. Informal caregivers and detection of delirium in postacute care: a correlational study of the confusion assessment method (CAM), confusion assessment method-family assessment method (CAM-FAM) and DSM-IV criteria. Int J Older People Nurs 2016;11:176–83. 26669904 10.1111/opn.12106

[14] Steis MR, Evans L, Hirschman KB, Hanlon A, Fick DM, Flanagan N, Screening for delirium using family caregivers: convergent validity of the family confusion assessment method and interviewer-rated confusion assessment method. J Am Geriatr Soc 2012;60:2121–6. 23039310 10.1111/j.1532-5415.2012.04200.x PMC3498543

[15] Mailhot T, Darling C, Ela J, Malyuta Y, Inouye SK, Saczynski J. Family identification of delirium in the emergency Department in Patients with and without Dementia: validity of the family confusion assessment method (FAM-CAM). J Am Geriatr Soc 2020;68:983–90. 32274799 10.1111/jgs.16438 PMC7370702

[16] Martins S, Conceição F, Paiva JA, Simões MR, Fernandes L. Delirium recognition by family: European Portuguese validation study of the family confusion assessment method. J Am Geriatr Soc 2014;62:1748–52. 25039562 10.1111/jgs.12973

[17] Bull MJ, Boaz L, Maadooliat M, Hagle ME, Gettrust L, Greene MT, Preparing family caregivers to recognize delirium symptoms in older adults after elective hip or knee arthroplasty. J Am Geriatr Soc 2017;65:e13–7. 27861701 10.1111/jgs.14535

[18] Greindl S, Weiss B, Magnolini R, Lingg C, Mayer H, Schaller SJ. Detection of delirium by family members in the intensive care unit: translation, cross-cultural adaptation and validation of the family confusion assessment method for the German-speaking area. J Adv Nurs 2022;78:3207–16. 35301750 10.1111/jan.15227

[19] Fiest KM, Krewulak KD, Ely EW, Davidson JE, Ismail Z, Sept BG, Partnering with family members to detect delirium in critically ill patients. Crit Care Med 2020;48:954–61. 32332281 10.1097/CCM.0000000000004367

[20] Zhou C, Wang H, Wang L, Zhou Y, Wu Q. Diagnostic accuracy of the family confusion assessment method for delirium detection: a systematic review and meta-analysis. J Am Geriatr Soc 2024;72:892–902. 38018490 10.1111/jgs.18692

[21] Aya AGM, Pouchain P-H, Thomas H, Ripart J, Cuvillon P. Incidence of postoperative delirium in elderly ambulatory patients: a prospective evaluation using the FAM-CAM instrument. J Clin Anesth 2019;53:35–8. 30292069 10.1016/j.jclinane.2018.09.034

[22] Paulson CM, Monroe T, McDougall GJ Jr, Fick DM. A family-focused delirium educational initiative with practice and research implications. Gerontol Geriatr Educ 2016;37:4–11. 26165565 10.1080/02701960.2015.1031896 PMC4708000

[23] Yan E, Veitch M, Saripella A, Alhamdah Y, Butris N, Tang-Wai DF, Association between postoperative delirium and adverse outcomes in older surgical patients: a systematic review and meta-analysis. J Clin Anesth 2023;90:111221. 37515876 10.1016/j.jclinane.2023.111221

[24] Avidan MS, Fritz BA, Maybrier HR, Muench MR, Escallier KE, Chen Y, The prevention of delirium and complications associated with surgical treatments (PODCAST) study: protocol for an international multicentre randomised controlled trial. BMJ Open 2014;4:e005651. 10.1136/bmjopen-2014-005651 PMC4166247 25231491

[25] Wildes TS, Winter AC, Maybrier HR, Mickle AM, Lenze EJ, Stark S, Protocol for the electroencephalography guidance of anesthesia to alleviate geriatric syndromes (ENGAGES) study: a pragmatic, randomised clinical trial. BMJ Open 2016;6:e011505. 10.1136/bmjopen-2016-011505 PMC4916634 27311914

[26] Wildes TS, Mickle AM, Ben Abdallah A, Maybrier HR, Oberhaus J, Budelier TP, Effect of electroencephalography-guided anesthetic administration on postoperative delirium among older adults undergoing major surgery: the ENGAGES randomized clinical trial. JAMA 2019;321:473–83. 30721296 10.1001/jama.2018.22005 PMC6439616

[27] Avidan MS, Maybrier HR, Abdallah AB, Jacobsohn E, Vlisides PE, Pryor KO, Intraoperative ketamine for prevention of postoperative delirium or pain after major surgery in older adults: an international, multicentre, double-blind, randomised clinical trial. Lancet 2017;390:267–75. 28576285 10.1016/S0140-6736(17)31467-8 PMC5644286

[28] Cuschieri S The STROBE guidelines. Saudi J Anaesth 2019;13:S31–4. 30930717 10.4103/sja.SJA_543_18 PMC6398292

[29] Maybrier HR, Mickle AM, Escallier KE, Lin N, Schmitt EM, Upadhyayula RT, Reliability and accuracy of delirium assessments among investigators at multiple international centres. BMJ Open 2018;8:e023137. 10.1136/bmjopen-2018-023137 PMC6252643 30467132

[30] Inouye SK, Leo-Summers L, Zhang Y, Bogardus ST Jr, Leslie DL, Agostini JV. A chart-based method for identification of delirium: validation compared with interviewer ratings using the confusion assessment method. J Am Geriatr Soc 2005;53:312–8. 15673358 10.1111/j.1532-5415.2005.53120.x

[31] Saczynski JS, Kosar CM, Xu G. A tale ofTwo methods: chart and interview methods for identifying delirium. J AmGeriatr Soc 2014;62:518–24. 10.1111/jgs.12684 PMC3959564 24512042

[32] Schmitt EM, Marcantonio ER, Alsop DC, Jones RN, Rogers SO Jr, Fong TG, Novel risk markers and long-term outcomes of delirium: the successful aging after elective surgery (SAGES) study design and methods. J Am Med Dir Assoc 2012;13(818):e1–10. 10.1016/j.jamda.2012.08.004 PMC3489992 22999782

[33] Baten V, Busch H-J, Busche C, Schmid B, Heupel-Reuter M, Perlov E, Validation of the brief confusion assessment method for screening delirium in elderly medical patients in a German emergency department. Acad Emerg Med 2018;25:1251–62. 29738102 10.1111/acem.13449

[34] Inouye SK, Dyck CHV, Alessi CA, Balkin S, Siegal AP, Horwitz RI. Clarifying confusion: the confusion assessment method. A new method for detection of delirium. Ann Intern Med 1990;113:941–8. 2240918 10.7326/0003-4819-113-12-941

[35] Han JH, Wilson A, Graves AJ, Shintani A, Schnelle JF, Dittus RS, Validation of the confusion assessment method for the intensive care unit in older emergency department patients. Acad Emerg Med 2014;21:180–7. 24673674 10.1111/acem.12309 PMC4034173

[36] Ely EW, Inouye SK, Bernard GR, Gordon S, Francis J, May L, Delirium in mechanically ventilated patients: validity and reliability of the confusion assessment method for the intensive care unit (CAM-ICU). JAMA 2001;286:2703–10. 11730446 10.1001/jama.286.21.2703

[37] Wei LA, Fearing MA, Sternberg EJ, Inouye SK. The confusion assessment method: a systematic review of current usage. J Am Geriatr Soc 2008;56:823–30. 18384586 10.1111/j.1532-5415.2008.01674.x PMC2585541

[38] Wang W, Lin N, Oberhaus JD, Avidan MS. Assessing method agreement for paired repeated binary measurements administered by multiple raters. Stat Med 2020;39:279–93. 31788847 10.1002/sim.8398 PMC7233794

[39] Zhou X-H, Obuchowski NA, McClish DK. Statistical methods in diagnostic medicine. John Wiley & Sons; 2014.

[40] Waikar SS, Betensky RA, Emerson SC, Bonventre JV. Imperfect gold standards for biomarker evaluation. Clin Trials 2013;10:696–700. 24006246 10.1177/1740774513497540 PMC3800226

[41] U.S. Department of Health and Human Services. Statistical guidance on reporting results from studies evaluating diagnostic tests. US Food and Drug Administration; 2007. https://www.fda.gov/regulatory-information/search-fda-guidance-documents/statistical-guidance-reporting-results-studies-evaluating-diagnostic-tests-guidance-industry-and-fda.

[42] Inouye SK, Marcantonio ER, Kosar CM, Tommet D, Schmitt EM, Travison TG, The short-term and long-term relationship between delirium and cognitive trajectory in older surgical patients. Alzheimers Dement 2016;12:766–75. 27103261 10.1016/j.jalz.2016.03.005 PMC4947419

[43] Shi Q, Warren L, Saposnik G, Macdermid JC. Confusion assessment method: a systematic review and meta-analysis of diagnostic accuracy. Neuropsychiatr Dis Treat 2013;9:1359–70. 24092976 10.2147/NDT.S49520 PMC3788697

[44] Bannon L, McGaughey J, Clarke M, McAuley DF, Blackwood B. Designing a nurse-delivered delirium bundle: what intensive care unit staff, survivors, and their families think? Aust Crit Care 2018;31:174–9. 29580965 10.1016/j.aucc.2018.02.007

[45] Rosenbloom-Brunton DA, Henneman EA, Inouye SK. Feasibility of family participation in a delirium prevention program for hospitalized older adults. J Gerontol Nurs 2010;36:22–33. quiz 34–5. 10.3928/00989134-20100330-02 PMC3666867 20438016

[46] Steffens NM, Tucholka JL, Nabozny MJ, Schmick AE, Brasel KJ, Schwarze ML. Engaging patients, health care professionals, and community members to improve preoperative decision making for older adults facing high-risk surgery. JAMA Surg 2016;151:938–45. 27368074 10.1001/jamasurg.2016.1308 PMC5071104

[47] Malley AM, Bourbonniere M, Naylor M. A qualitative study of older adults’ and family caregivers’ perspectives regarding their preoperative care transitions. J Clin Nurs 2018;27:2953–62. 29633436 10.1111/jocn.14377 PMC6545899

[48] Smithburger PL, Korenoski AS, Kane-Gill SL, Alexander SA. Perceptions of family members, nurses, and physicians on involving patients’ families in delirium prevention. Crit Care Nurse 2017;37:48–57. 29196587 10.4037/ccn2017901

