# Supplementary material for: Detection of postoperative delirium by family and caregivers: Evaluation of the family confusion assessment method (FAM-CAM)
Source: J Clin Anesth. Author manuscript; Available in PMC 2025 Sep 9. (PMC12419121; doi:10.1016/j.jclinane.2025.111963)
Supplement: MMC1 [file NIHMS2107626-supplement-MMC1.pdf]

# Method agreement assessment for CAM and FAM-CAM

## 1 Methodology

We label the two methods CAM and FAM-CAM as 'C' and 'F', respectively. Let $(y_{ijtC}, y_{ij'tF})$ denote the pair of binary measurements from CAM and FAM-CAM on a randomly selected subject $i$ at time point $t$ recorded at the same time by two different raters $j$ and $j'$ randomly selected from a population of raters. To model the binary measurements (either 0 or 1), we use a generalized linear mixed model (GLMM) with a probit link function, given by

$$y_{ijtm}|\gamma_i, \alpha_{jm} \sim \text{Bernoulli}(\pi_{ijtm}),$$

and

$$\pi_{ijtm} = \Phi(\mu_{ijtm}),$$

where $m \in \{C, F\}$, $i = 1, 2, \ldots, I$, $j = 1, 2, \ldots, J$, $t = 1, \ldots, T_i$, and $\Phi(\cdot)$ is the cdf of the standard normal. The linear predictor $\mu_{ijtm}$ is given as

$$\mu_{ijtm} = \beta_m + t + \gamma_i + \alpha_{jm}. \tag{1.1}$$

Terms in (1.1) are assumed as follows.

- $\beta_m$ is the fixed effect of Method $m$, $m \in \{C, F\}$;
- The regression on time $t$ allows to incorporate longitudinal measurements on subjects when the mean response value changes over time.
- $\gamma_i$ is the random effect of subjects, and $\gamma_i \overset{iid}{\sim} N(0, \sigma^2_\gamma)$;
- $\alpha_{jm}$ is the random effect of raters within Method $m$, and $\alpha_{jm} \overset{iid}{\sim} N(0, \sigma^2_{\alpha m})$.

Notice the dependence of the rater's variance components on $m$, we allow the rater's random effects to vary across different methods, while the subject's random effects remain the same across methods.

## 2 Analysis and Results

### 2.1 GLMM

GLIMMIX procedure in SAS is used to analyze the data based on the generalized linear mixed model proposed.

Table 1: Test for method agreement

| Effect | NumDF | DenDF | FValue | ProbF |
|---|---|---|---|---|
| method | 1 | 12.74 | 9.31 | 0.0095 |

Table 2: Estimates of variance components

| CovParm | Subject | Group | Estimate | StdErr |
|---|---|---|---|---|
| id | | | 1.4037 | 0.09881 |
| rater1 | | method C | 0.1590 | 0.08718 |
| rater1 | | method F | 0.03745 | 0.04355 |
| AR(1) | id(method) | | 0.03101 | 0.03484 |
| Residual | | | 0.3522 | 0.01151 |

Table 1 shows the $p$-value for testing $H_0 : \beta_C - \beta_F = 0$, is $0.0095 < 0.05$. Thus, at level 0.05, we reject $H_0$, and hence we can conclude that the two methods disagree.

Table 2 shows the estimates of variance components:

$$\hat{\sigma}_\gamma^2 = 1.4037, \quad \hat{\sigma}_{\alpha C}^2 = 0.1590, \text{ and } \hat{\sigma}_{\alpha F}^2 = 0.03745.$$

To check the inter-rater reliability, we calculate the ICCs for both methods. The $\text{ICC}_{CAM}$ for method CAM is given as

$$\frac{\hat{\sigma}_\gamma^2 + 1}{\hat{\sigma}_\gamma^2 + \hat{\sigma}_{\alpha_C}^2 + 1} = 0.938.$$

The $\text{ICC}_{FAM}$ for method DCAM is given as

$$\frac{\hat{\sigma}_\gamma^2 + 1}{\hat{\sigma}_\gamma^2 + \hat{\sigma}_{\alpha_F}^2 + 1} = 0.985.$$

Thus, for both methods, raters had an excellent degree of agreement.

## 2.2 Bland-Altman Plot

Figure 1 shows the Bland-Altman plots.

For the Z-score scaled Bland-Altman plot,

- Each '+' represents a patient. The x-axis is the average of Z-scores of the event 'score=1' measured by CAM and FAM-CAM. The y-axis is the difference between Z-scores of the event 'score=1' measured by CAM and FAM-CAM.
- The paired green dashed lines plot the 95% agreement limits, which covers the zero difference. As expected, 95.9% of the points lie within $\pm 2(sd)$ of the mean difference.
- The mean difference is -0.48, which indicates on average, the Z-score by CAM is 0.48 lower than that by FAM-CAM.

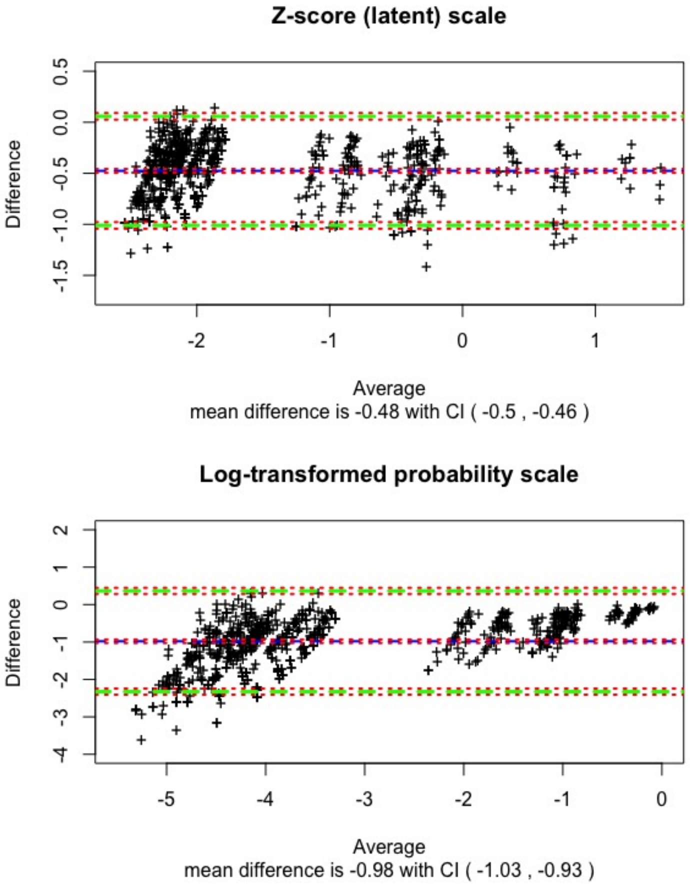


Figure 1: Bland Altman Plot on Z-score scale

For the log-transformed probability scaled Bland-Altman plot,

- Each '+' represents a patient. The x-axis is the average of log( probabilities of the event 'score=1') measured by CAM and FAM-CAM. The y-axis is the difference between log( probabilities of the event 'score=1') measured by CAM and FAM-CAM. For example, a point in the plot with y-value $-0.1$ means, for that patient, log( probabilities of the event 'score=1') based on method CAM is 0.1 lower than that based on method FAM-CAM. In other words, the ratio of probabilities of the event 'score=1' between CAM and FAM-CAM is $e^{-0.1} = 0.9$.
- The paired green dashed lines plot the 95% agreement limits, which also covers the zero difference. As expected, 95.4% of the points lie within $\pm 2(sd)$ of the mean difference.
- The blue dashed line shows the mean difference. The mean difference is -0.98, which indicates that the probability of score '1' measured by FAM-CAM is on average $e^{0.98} = 2.7$ times the probability by CAM.

## 2.3 Cohen's Kappa

The predicted 0-1 binary score of the $i$th patient by method $m$ is defined as

$$\hat{y}_{im} = \begin{cases} 1, & \text{if } \hat{\mu}_{im} > 0, \\ 0, & \text{o.w.} \end{cases}$$

Here, $\hat{\mu}_{im}$ is the EBLUP of $\overline{\mu}_{im}$ defined as

$$\overline{\mu}_{im} = \beta_m + \frac{1}{T_i}\sum_{t=1}^{T_i} t + \frac{J\sigma_\gamma^2}{J\sigma_\gamma^2 + \sigma_{\alpha m}^2}\left(\gamma_i + \frac{1}{J}\sum_{j=1}^{J}\alpha_{jm}\right). \qquad (2.1)$$

Suppose that the disagreement count data are as follows:

| | CAM=0 | CAM=1 |
|---|---|---|
| FAM-CAM=0 | a | b |
| FAM-CAM=1 | c | d |

Then, Cohen's Kappa $\kappa$ is given by

$$\kappa = \frac{p_o - p_e}{1 - p_e},$$

where

$$p_o = \frac{a+d}{a+b+c+d},$$

$$p_e = \frac{a+b}{a+b+c+d} \cdot \frac{a+c}{a+b+c+d} + \frac{c+d}{a+b+c+d} \cdot \frac{b+d}{a+b+c+d}.$$

For our data, Cohen's Kappa $\kappa$ calculated by the R software is 0.72, with the 95% confidence interval [0.63,0.81], which indicates a good agreement.

# 3 Conclusion

- We analyze the agreement of CAM and FAM-CAM by the generalized linear mixed model (GLMM). Since the response variable is binary ( 0 or 1), we use probit link function. Since each patient may have repeated measurements, we consider R-side AR(1) GLMM. The linear predictor of the model consists of a fixed intercept, fixed method effects, and random effects from patients and raters. Based on the hypothesis testing for the difference between fixed effects of two methods, we reject the null hypothesis that the fixed effects of two methods are the same. Therefore, the two methods disagree.

- The Bland Altman plot gives a visual impression that CAM has lower probability of the event 'score=1' compared to FAM-CAM. In detail, the probability of score '1' measured by FAM-CAM is on average 2.7 times the probability by CAM.

- Cohen's Kappa is 0.72, with the 95% confidence interval [0.63,0.81], which indicates a good agreement.
